# Analysis of the Surface Morphology of Dental Enamel Exposed to Ready‐to‐Drink Alcoholic Beverages

**DOI:** 10.1155/ijod/9930736

**Published:** 2026-01-16

**Authors:** Marcel Alves Avelino de Paiva, Anderson Gomes Forte, Juliellen Luiz da Cunha, Paulo Vitor de Souza Silva, Elizabeth Barreto Galvão de Sousa, Andressa Feitosa Bezerra de Oliveira

**Affiliations:** ^1^ Post-Graduate Program in Dentistry, Health Science Center, Federal University of Paraiba (UFPB), João Pessoa, Paraíba, Brazil, ufpb.br; ^2^ Post-Graduate Program in Dental Materials, Piracicaba School of Dentistry – Unicamp, Piracicaba, São Paulo, Brazil; ^3^ Post-Graduate Program in Public Health, Health Science Center, Federal University of Rio Grande do Norte (UFRN), Natal, Rio Grande do Norte, Brazil, ufrn.br; ^4^ Department of Morphology, Health Science Center, Federal University of Paraiba, João Pessoa, Paraíba, Brazil, ufpb.br

**Keywords:** alcohol drinking, tooth demineralization, tooth erosion

## Abstract

**Background:**

Ready‐to‐drink (RTD) alcoholic beverages often contain organic acids that lower pH and promote enamel demineralization, yet their erosive potential remains insufficiently explored.

**Objective:**

This study aimed to evaluate the erosive potential of RTDs through chemical characterization and analysis of enamel surface alterations.

**Methods:**

Sixty bovine enamel blocks were divided into six groups (*n* = 10): Ice Smirnoff, Skol Beats Senses, Schweppes Vodka Citrus, Jack Daniel’s & Coca‐Cola, Coca‐Cola (positive control), and mineral water (negative control). The beverages were analyzed for pH, titratable acidity (TA), and buffering capacity (*β*). Enamel alterations were assessed using surface microhardness (SMH), while surface roughness (Sa) and enamel surface loss (SL) were measured by optical 3D profilometry. Specimens were immersed in each beverage for 120 min under controlled laboratory conditions with gentle agitation. Data were analyzed using ANOVA and Tukey’s post hoc test (*α* = 0.05).

**Results:**

All RTD beverages exhibited acidic pH (2.75–3.04). Ice Smirnoff and Skol Beats Senses showed the highest TA and *β* values. Except for the negative control, all beverages significantly reduced SMH, increased Sa, and presented significant enamel SL (*p*  < 0.05). The severity of erosive changes was strongly associated with beverage composition, particularly citric acid content and *β*.

**Conclusion:**

RTD alcoholic beverages demonstrated significant erosive potential, promoting enamel surface softening, increased roughness, and SL.

## 1. Introduction

Dental erosion is defined as a pathological, chronic, and localized loss of hard dental tissue caused by exposure to acids of intrinsic or extrinsic origin, leading to progressive mineral dissolution and compromise of enamel integrity [1]. Hydroxyapatite, the primary mineral component of enamel, becomes increasingly soluble in acidic environments, promoting demineralization, hardness loss, and enamel softening, which makes tissue more susceptible to mechanical wear [2]. Although softened enamel may undergo remineralization in the presence of calcium‐rich and phosphate‐rich saliva, erosive wear is often asymptomatic and progresses slowly, delaying patient perception and treatment seeking [3].

In recent years, the prevalence of dental erosion has increased among adolescents and young adults, associated with conditions such as bulimia, gastroesophageal reflux, and especially the high consumption of acidic foods and beverages [4–7]. Parallel to these developments, the alcoholic beverage industry has expanded the production of ready‐to‐drink (RTD) products, which combine alcohol with sugary or flavored diluents to meet rising consumer demand [8, 9]. According to the WHO Global Status Report on Alcohol and Health [10], 2.5 billion people over the age of 15 consumed alcoholic beverages in 2019, with Europe and the Americas presenting the highest per capita intake. These data underscore the relevance of understanding consumption patterns and their public health implications.

RTD beverages typically contain organic acids such as citric, malic, and phosphoric acids, which can promote enamel mineral dissolution through gradual dissociation [1, 2, 11]. Unlike caries, which results from bacterial acid production and primarily affects biofilm‐covered areas, dental erosion is a surface phenomenon caused by direct chemical attack [11]. Repeated acid exposure leads to enamel softening and may favor the progression of erosive wear [1, 12].

Despite advances in industrial safety regulations, the popularity of RTD beverages has grown considerably among younger consumers, particularly older adolescents and young adults [8, 13–15]. These products pose the same health risks as other alcoholic drinks and may additionally affect oral health due to their acidity and overall chemical composition [16–19]. Reports from Euromonitor International [20] highlight the expansion and diversification of the RTD market, driven by beverages inspired by popular cocktails, such as Schweppes Gin Tonic Spritz and Caipi Beats by Coca‐Cola. Although global consumption continues to rise [10], market analyses indicate that growth in Brazil has been more gradual [20].

Despite the well‐established knowledge on the erosive effects of acidic beverages, evidence specifically addressing the erosive potential of RTD alcoholic beverages remains limited. These products differ from traditional soft drinks and conventional alcoholic beverages because their formulations combine alcohol, organic acids, flavoring agents, carbonation, and variable sugar content—factors that may influence erosive behavior in distinct ways [9]. Given the rapid expansion of the RTD market and the limited experimental evidence available, further research is needed.

Given their increasing consumption among younger populations, assessing the erosive potential of RTD alcoholic beverages is highly relevant. Therefore, this in vitro study aimed to evaluate the erosive effects of RTD beverages by assessing their pH, titratable acidity (TA), and buffering capacity (*β*), as well as enamel surface alterations measured through microhardness, surface roughness (Sa), and surface loss (SL). The null hypothesis was that RTD alcoholic beverages would not significantly affect the surface morphology or mechanical properties of dental enamel.

## 2. Materials and Methods

### 2.1. Experimental Design

The study was conducted in two distinct phases. In the first phase, the chemical properties of all beverages were measured, including pH, TA, and *β*. In the second phase, the beverages were evaluated for their erosive effect on enamel (*n* = 10 per group) using an adapted erosive challenge model described by Silva et al. [21]. The experimental design was randomized, with a single factor considered in all tests. The response variable for Phase 1 was the volume (mL) of 1M NaOH required for titration. In Phase 2, the response variables were the reduction in surface microhardness (SMH), the increase in Sa, and enamel SL measured by 3D optical profilometry.

### 2.2. Sample Size Calculation and Specimen Preparation

The sample size calculation was performed to detect a minimum difference of 12.5% in surface microhardness loss between groups, assuming a standard deviation of 7.5%, a significance level of 5%, and a statistical power of 80%. These parameters were based on previous studies reporting similar microhardness variability [18, 21]. The calculation followed the formula*n =* 2 (Zα/2 + Zβ)^2^ SD^2^/*d* ^2^, where Zα/2 = 1.96 (5% significance), Zβ = 0.84 (80% power), SD = 7.5, and *d* = 12.5. This resulted in a minimum of six specimens per group. To account for possible experimental losses and ensure measurement reliability, the number was increased to 10 specimens per group.

A total of 35 bovine incisor teeth were collected from government‐inspected slaughterhouses, with no animals sacrificed specifically for research. All procedures involving animal‐derived material complied with institutional and international ethical standards. The teeth were stored in 0.08% thymol solution at room temperature for up to 30 days until specimen preparation. From these, 60 enamel blocks free of cracks, fractures, or structural defects were prepared (5 × 5 × 2 mm) using a precision diamond saw (Labcut 1010, Extec Corp., Enfield, CT, USA) under constant irrigation. The specimens were embedded in self‐curing acrylic resin using 16‐mm diameter molds (3 mm depth). The enamel surfaces were flattened with silicon carbide papers (600–1500 grit) under water cooling and polished with 1 µm diamond paste (Extec Corporation, Enfield, CT) using a rotary polishing unit (PSK‐2V, Skill‐Tec Trade, São Paulo, Brazil).

Specimens were randomly allocated to the experimental groups (*n* = 10) based on the initial SMH (SMH_0_) measurements. Each enamel surface was subdivided into three regions: the two outer thirds were protected with two layers of nail varnish (Risqué, Niasi, São Paulo, Brazil), leaving a central window of approximately 1 mm^2^ exposed for the erosive challenge.

### 2.3. Selection of Beverages

The RTD alcoholic beverages evaluated in this study were purchased from local supermarkets according to their seasonal availability and allocated into four experimental groups, as described in Table 1. All beverages were stored and handled in accordance with the manufacturers’ recommendations to ensure the preservation of their original chemical properties.

**Table 1:** Ready‐to‐drink alcoholic beverages and control solutions tested in the study.

Beverage group	Composition	Manufacturer (batch)
Ice Smirnoff	Carbonated water; sake; sugar; ethyl alcohol; acidulants (citric, tartaric, and malic acids); sodium citrate; sodium benzoate. Alcohol content: 5%.	Diageo (L3320J6013)
Skol Beats Senses	Carbonated water; dry sake (fermented rice alcohol and ethyl alcohol); sugar; acidulants (citric and tartaric acids); lemon flavoring; sodium citrate; malic acid. Alcohol content: 7.9%.	Skol (NS08092B)
Schweppes Vodka Citrus	Carbonated water; sake; sugar; vodka; acidulant (citric acid); sodium benzoate; sodium sorbate; potassium sorbate. Alcohol content: 5%.	Coca‐Cola (P281223)
Jack Daniel’s & Coca‐Cola	Carbonated water; Tennessee whiskey; sugar; kola nut extract; caramel coloring; acidulant (phosphoric acid). Alcohol content: 5%	Brown‐Forman/Coca‐Cola (P291023)
Mineral water (negative control)	Mineral salts (bicarbonate, calcium, magnesium, sodium, potassium, chloride, sulfate, fluoride), according to manufacturer labeling.	Minalba (L2460014)
Coca‐Cola (positive control)	Carbonated water; sugar; kola nut extract; caffeine; caramel coloring; acidulant (phosphoric acid).	Coca‐Cola (P080624)

*Note:* Composition information was extracted from manufacturer labeling.

### 2.4. Determination of pH, TA, and *β*

Immediately after opening, the pH of each beverage was measured using an electrode coupled to a pH meter (Orion 290A+, Thermo Electron Corporation). TA was determined by adding 0.2 mL increments of 1M NaOH solution to 50 mL of each beverage and recording the volume required to reach pH 5.5 and 7.0. All measurements were performed at room temperature (22–25°C) under constant agitation. Each beverage was analyzed in triplicate, and mean values were used for statistical analysis. *β* was calculated according to the method described by Lussi et al. [22], using the formula:

β=ΔC/ΔpH,

where Δ*C* represents the amount of titrant added and ΔpH is the resulting change in pH.

To minimize potential bias, all beverages were transferred to coded containers by an independent researcher before analysis, ensuring that their identities remained concealed throughout the experimental procedures.

### 2.5. *E*rosive Challenge

The enamel specimens were immersed in 50 mL of each beverage (5.56 mL of solution per mm^2^ of exposed enamel surface) at room temperature (22–25°C) for 120 min under gentle agitation [21]. After the erosive challenge, specimens were rinsed with deionized water, sonicated for 5 min, and stored in a controlled‐humidity environment until further analysis.

This erosive challenge followed a continuous immersion model adapted from Silva et al. [21]. Unlike cyclic erosive protocols that alternate acidic and remineralizing phases, the continuous immersion approach maintains constant acid exposure, allowing the isolated evaluation of the intrinsic chemical erosive potential of the beverages. A standardized volume‐to‐surface ratio and gentle agitation were used, following procedures widely adopted in mechanistic in vitro erosion studies.

### 2.6. SMH Determination

SMH_0_ was measured using a microhardness tester (HMV‐G21, Shimadzu, Kyoto, Japan) equipped with a Vickers diamond indenter. A 50‐g load was applied for 10 s, producing three indentations spaced 100 µm apart. After the erosive challenge, final surface microhardness (SMH_1_) was measured using the same parameters. The percentage change in surface microhardness (%SMHC) was calculated using the formula:

%SMHC=SMH0−SMH1 SMH0×100,

where SMH_0_ represents the initial surface microhardness and SMH_1_ the final surface microhardness.

### 2.7. Surface Morphology Analysis of Enamel (Sa and SL)

Sa and enamel SL were measured using a three‐dimensional noncontact optical profilometer (TALY‐SURF CCI MP, Taylor Hobson, Leicester, England). The equipment was set with a cutoff of 0.25 mm, a 10× lens, a numerical aperture of 0.4, and a scanning speed of 1× in “XY” mode. A surface area of approximately 1.7 × 1.7 mm was scanned, encompassing both sound and eroded surfaces.

Sa was calculated separately for the sound and eroded areas, and surface roughness variation (ΔSa) was obtained using the formula:

ΔSa=SaE−SaH,

where SaE is the roughness of the eroded area and SaH the roughness of the sound area.

Enamel SL was quantified by drawing linear profiles between sound and eroded areas at three vertical positions of the specimen: 75% (upper third), 50% (middle third), and 25% (lower third). At each level, SL was determined by subtracting the height of the sound surface from that of the eroded surface. The mean of the three measurements was used as the final SL value for each specimen.

In addition to quantitative analyses, three‐dimensional surface images were acquired to qualitatively illustrate changes in roughness and SL produced by the alcoholic beverages and control solutions.

### 2.8. Statistical Analysis

Data normality was assessed using the Shapiro–Wilk test, and homogeneity of variances was verified using Levene’s test. Because the data met the assumptions of normality and homoscedasticity, one‐way ANOVA was applied to compare differences among groups, followed by Tukey’s post hoc test for multiple comparisons. Effect sizes were calculated using the omega‐squared (*ω* ^2^) coefficient to estimate the magnitude of group effects beyond statistical significance.

The variables analyzed included pH, TA, *β*, SMH_0_ and SMH_1_, %SMHC, ΔSa, and enamel SL. These parameters were selected to characterize the chemical properties of the beverages and their effects on enamel morphology.

The significance level was set at 5% (*p*  < 0.05). All analyses were performed using SPSS software, version 21 (IBM Corp., Armonk, NY, USA).

## 3. Results

The present study evaluated the erosive potential of four RTD alcoholic beverages in comparison with a positive control (Coca‐Cola) and a negative control (mineral water). Chemical characteristics (pH, TA, *β*) and enamel alterations (surface microhardness, Sa, and enamel SL) were analyzed to characterize the interaction between these beverages and dental enamel.

The chemical properties of the beverages are presented in Table 2. All RTD drinks exhibited pH values below the critical limit of 5.5 for enamel dissolution. Based on the classification proposed by Larsen and Nyvad [23], Jack Daniel’s & Coca‐Cola, Schweppes Vodka Citrus, and Skol Beats Senses were categorized as highly erosive (pH 2.00–3.00), whereas Ice Smirnoff was classified as erosive (pH 3.00–4.00). TA and *β* also varied significantly among beverages. Ice Smirnoff required the greatest volume of NaOH to reach neutral pH, exhibiting the highest TA and *β*, followed by Skol Beats Senses and Schweppes Vodka Citrus. In contrast, Jack Daniel’s & Coca‐Cola showed the lowest TA and buffering effect among the acidic beverages. The negative control (mineral water) did not undergo titration because its initial pH was above neutrality. Effect size analysis revealed extremely high *ω* ^2^ values (0.994–0.999), indicating that the type of beverage accounted for nearly all variability in pH, TA, and *β*, confirming pronounced chemical differences between groups.

**Table 2:** Mean (standard deviation) of initial pH values, titratable acidity (TA) to pH 5.5 and 7.0, and buffering capacity (*β*) of the analyzed beverages.

Beverage group	Initial pH	TA		*β*
		pH 5.5	pH 7.0	
Ice Smirnoff	3.04 (0.01)^c^	3.88 (0.05)^b^	5.76 (0.17)^b^	26.13 (0.71)^b^
Skol Beats Senses	2.87 (0.02)^d^	3.50 (0.05)^c^	5.50 (0.01)^b^	24.01 (0.15)^c^
Schweppes Vodka Citrus	2.73 (0.01)^e^	1.80 (0.05)^d^	3.37 (0.20)^c^	14.76 (0.85)^d^
Jack Daniel’s & Coca‐Cola	2.35 (0.02)^f^	0.65 (0.00)^a^	1.75 (0.05)^a^	7.26 (0.19)^a^
Negative control	7.54 (0.08)^b^	—	—	—
Positive control	2.57 (0.01)^a^	0.57 (0.02)^a^	1.77 (0.02)^a^	7.69 (0.10)^a^
Effect size (*ω* ^2^)	0.997	0.999	0.994	0.995

*Note:* Effect size was calculated using *ω* ^2^. Lowercase letters in the same column indicate statistically significant differences between groups (one‐way ANOVA, Tukey’s test, *p* < 0.05).

SMH₀ and SMH_1_ values, together with %SMHC, are shown in Table 3. Except for the negative control, all beverages significantly reduce enamel microhardness. Ice Smirnoff, Skol Beats Senses, and Schweppes Vodka Citrus produced the greatest reduction in surface microhardness, with percentage loss values significantly higher than the positive control. Jack Daniel’s & Coca‐Cola resulted in the lowest microhardness reduction among the RTD beverages, with values comparable to the positive control. Effect size values for SMH_1_ and %SMHC were extremely high (*ω* ^2^ = 0.970 and 0.969), indicating that beverage type explained nearly all microhardness variability.

**Table 3:** Mean (standard deviation) values of initial surface microhardness (SMH_0_), final microhardness (SMH_1_), and percentage change in surface microhardness (%SMHC) for all groups.

Beverage group	SMH_0_	SMH_1_	% SMHC
Ice Smirnoff	385.35 (6.85)^a^	105.12 (19.71)^c^	72.68 (5.28)^c^
Skol Beats Senses	387.86 (6.21)^a^	107.40 (10.35)^c^	72.30 (2.66)^c^
Schweppes Vodka Citrus	383.66 (4.77)^a^	104.90 (21.54)^c^	72.65 (5.60)^c^
Jack Daniel’s & Coca‐Cola	383.36 (6.57)^a^	133.05 (18.96)^b^	65.28 (4.98)^b^
Negative control	384.40 (5.66)^a^	380.60 (6.10)^a^	0.96 (2.37)^a^
Positive control	388.08 (5.27)^a^	135.90 (21.65)^b^	65.00 (5.40)^b^
Effect size (*ω* ^2^)	0.018	0.970	0.969

*Note:* Effect size was calculated using *ω* ^2^. Different lowercase letters in the same column indicate statistically significant differences between groups (one‐way ANOVA, Tukey, *p* < 0.05).

ΔSa and enamel SL is presented in Table 4. All RTD beverages caused an increase in Sa when compared with the negative and positive controls. Ice Smirnoff, Skol Beats Senses, and Schweppes Vodka Citrus produced significantly higher ΔSa values than the positive control, whereas Jack Daniel’s & Coca‐Cola exhibited the lowest increase in roughness among the RTDs. Enamel SL followed a similar pattern. Ice Smirnoff produced the greatest SL values, significantly higher than those of the other RTDs. Skol Beats Senses and Schweppes Vodka Citrus produced intermediate SL values, and Jack Daniel’s & Coca‐Cola again showed the lowest SL among the acidic beverages. The positive control exhibited moderate SL, while the negative control presented negligible alterations. Effect sizes confirmed these patterns, with *ω* ^2^ = 0.492 for ΔSa (moderate–high effect) and *ω* ^2^ = 0.910 for SL (very high effect), indicating that beverage type was the dominant determinant of erosive surface changes.

**Table 4:** Mean (standard deviation) values of surface roughness variation (ΔSa) and enamel surface loss (SL) for all groups.

Beverage group	∆Sa	SL
Ice Smirnoff	0.18 (0.11)^c^	6.36 (0.73)^a^
Skol Beats Senses	0.17 (0.11)^c^	5.23 (0.89)^c,d^
Schweppes Vodka Citrus	0.17 (0.13)^c^	4.55 (0.67)^c,e^
Jack Daniel’s & Coca‐Cola	0.09 (0.03)^a,c^	3.91 (0.72)^e^
Negative control	‐0.04 (0.02)^b^	0.12 (0.17)^b^
Positive control	0.06 (0.05)^a^	5.78 (0.53)^a,d^
Effect size (*ω* ^2^)	0.492	0.910

*Note:* Effect size was calculated using *ω* ^2^. Different lowercase letters in the same column indicate statistically significant differences between groups (one‐way ANOVA, Tukey, *p* < 0.05).

Figure 1 displays the qualitative topographic analysis of enamel surfaces. Colorimetric mapping of the eroded region (E) revealed clear differences in lesion depth across groups. Ice Smirnoff displayed predominantly bluish areas, indicating pronounced enamel loss. Skol Beats Senses and Schweppes Vodka Citrus exhibited greenish‐blue tones, reflecting moderate to severe erosion. Jack Daniel’s & Coca‐Cola showed mostly reddish and yellowish areas with limited green margins, suggesting shallower lesions. The negative control demonstrated uniform green and yellow areas consistent with an intact surface, whereas the positive control revealed notable wear characterized by predominantly green regions. These visual findings are consistent with the quantitative results and reinforce that RTD beverages with high TA, elevated *β*, and organic acids such as citric and malic acid exhibit substantial erosive potential on dental enamel.

Figure 1Topographic surface of intact (H) and eroded (E) dental enamel, obtained by 3D optical profilometry, after exposure to each evaluated beverage. (A) Ice Smirnoff; (B) Skol Beats Senses; (C) Schweppes Vodka Citrus; (D) Jack Daniel’s & Coca‐Cola; (E) Negative control; (F) Positive control.

**Figure:**
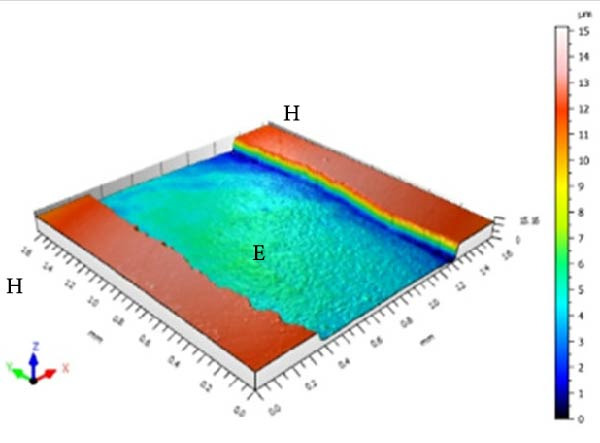
(A)

**Figure:**
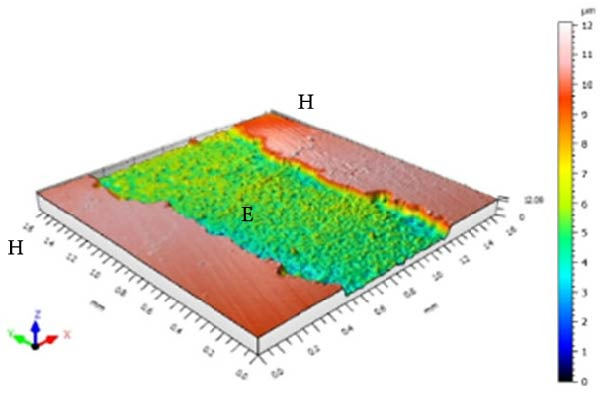
(B)

**Figure:**
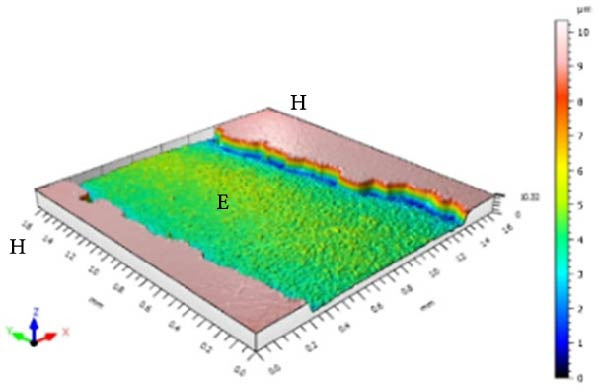
(C)

**Figure:**
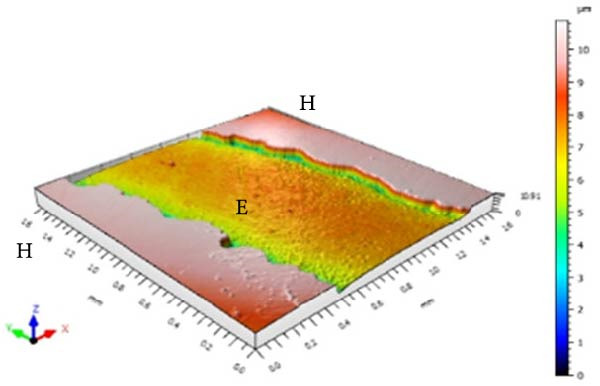
(D)

**Figure:**
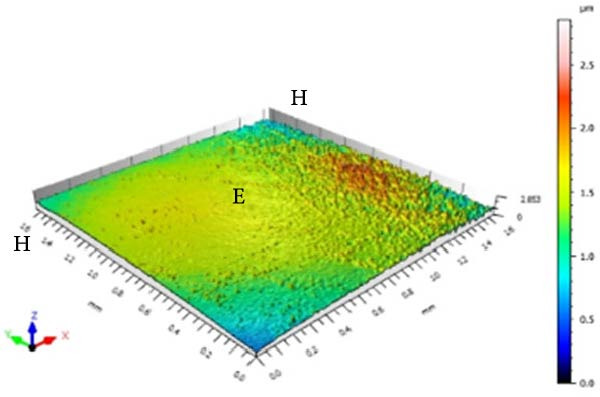
(E)

**Figure:**
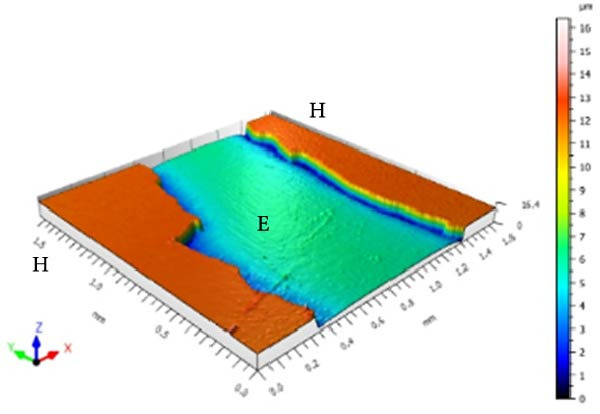
(F)

## 4. Discussion

The relationship between alcohol consumption and dental erosion remains controversial, and the specific mechanisms by which alcoholic beverages contribute to enamel degradation are not fully elucidated [24]. Previous studies indicate that acidity and beverage formulation, particularly acid type, TA, and *β*, play key roles in determining erosive potential [25–27].

According to Euromonitor International [20], the RTD alcoholic beverage market is highly diverse and continues to expand globally, especially among older adolescents and young adults [16]. These products commonly contain organic acids such as citric, tartaric, malic, acetic, and lactic acids, which enhance flavor but also contribute to acidity [26]. Because solutions with low pH and elevated TA and *β* tend to exhibit stronger demineralization potential [25], assessing these parameters becomes particularly important in erosion studies.

Consistent with findings by Lan et al. [24], Barac et al. [27], and Ablal et al. [19], all RTDs in the present study showed acidic pH values, a recognized prerequisite for dissolution of hydroxyapatite. However, pH alone does not accurately predict erosive potential, since mineral loss is also influenced by TA, *β*, acid type, and enamel supersaturation [2, 22, 23]. Ice Smirnoff, Skol Beats Senses, and Schweppes Vodka Citrus demonstrated the highest TA and *β* values, indicating prolonged acid availability and reduced neutralization capacity, factors that intensify demineralization [25]. Their citric acid content further increases aggressiveness because citric acid dissociates strongly and chelates calcium ions, destabilizing the enamel surface [19, 25, 26]. The presence of malic acid in some formulations may enhance these effects by reducing saturation with respect to enamel minerals [27]. These differences were supported by the large effect sizes, indicating that beverage composition accounted for nearly all variability in the chemical parameters.

Coca‐Cola, despite its low pH, displayed lower *β*, which helps explain why its erosive potential was similar or lower than some RTDs [28]. These chemical distinctions were clearly reflected in analytical measurements. Microhardness testing revealed significant enamel softening for all RTDs, especially those containing citric acid, in agreement with previous reports [24]. Sa analysis corroborated this pattern, demonstrating that beverages with high TA and complex acid mixtures produce more pronounced topographical alterations [18, 19, 25]. Optical profilometry showed actual enamel SL, capturing cumulative structural damage that may not be fully detected by microhardness or roughness alone [18]. Similar reductions in microhardness and increases in SL after exposure to acidic alcoholic beverages have been reported by Meira et al. [18], reinforcing the biological plausibility of the present results. The high effect sizes for SMH, roughness, and SL also indicate a consistent relationship between chemical acidity parameters and the extent of enamel degradation.

Surface microhardness was selected as a primary indicator of early mineral loss due to its sensitivity to detect initial enamel softening [18, 21, 24, 29]. However, post‐challenge microhardness values may reflect not only chemical softening but also the removal of softened enamel during drying or handling, contributing to measurement variability [30, 31]. Therefore, microhardness must be interpreted alongside complementary methods such as SL and Sa, which provide additional information on volumetric changes and topographical breakdown, as emphasized by Attin and Wegehaupt [32]. In this context, the combined assessment of microhardness, roughness and enamel SL provided a more comprehensive characterization of RTD‐induced erosion.

Unlike classical cyclic erosive protocols, which reproduce intraoral dynamics by alternating acid exposure, salivary buffering and pellicle reformation, continuous immersion models are used to isolate the intrinsic chemical erosive potential of test solutions [33]. By removing protective factors such as salivary proteins, pellicle formation, intermittent pH neutralization and mechanical buffering, these models increase sensitivity for detecting early mineral loss and enable clearer mechanistic comparisons among acidic solutions [34]. However, despite improving analytical sensitivity, continuous immersion does not replicate the complexity of in vivo conditions. This limitation should be considered when interpreting the magnitude of the observed SL, which likely represents an upper‐bound estimate under purely chemical conditions.

The use of bovine enamel further supports the reproducibility of this model. Recent evidence demonstrates that bovine and human enamel share similar prism morphology, calcium distribution, and responses to acid exposure, validating bovine enamel as a reliable substitute for standardized in vitro erosion protocols [35]. Thus, the enamel responses observed in this study likely reflect genuine substrate‐dependent erosive mechanisms rather than model‐related artifacts.

Among the evaluated beverages, Ice Smirnoff produced the most severe enamel alterations, consistent with its high *β* and strong organic acid profile [1, 18]. This was visually confirmed through profilometry, which showed deeper lesion areas, appearing in darker tones. Skol Beats Senses also exhibited substantial SL with heterogeneous lesion contours, likely due to its strong organic acid composition [36]. Schweppes Vodka Citrus caused moderate erosion, with less uniform and shallower lesions. In contrast, Jack Daniel’s & Coca‐Cola, despite its low pH, presented the lowest erosive effect among RTDs, consistent with its lower *β* and the more preserved enamel surface observed [1, 18]. These findings align with the well‐documented aggressive effects of citric acid reported in previous studies [18, 21, 29, 37].

Overall, the reductions in microhardness, increases in Sa and quantifiable enamel SL confirm that RTD alcoholic beverages exhibit significant erosive potential, in some cases comparable to or exceeding that of Coca‐Cola. The null hypothesis was therefore rejected.

Although in vitro models allow controlled quantification of mineral loss, they cannot reproduce the dynamic biological environment of the oral cavity [37]. Clinical studies consistently show associations between frequent acidic beverage consumption and erosive tooth wear, especially in adolescents and young adults [4, 5, 12]. Social drinking patterns involving acidic alcoholic beverages, such as wine, alcopops and mixed drinks, have also been identified as contributors to erosion [38]. While clinical evidence specifically focused on RTDs remains limited, current findings support the biological plausibility of their erosive potential.

In vivo, enamel is protected by several mechanisms absent in continuous immersion models. Saliva provides *β*, mineral ions, and organic components that modulate erosion [30, 39, 40]. Whole human saliva has been shown to offer substantially greater protection against erosion than dialyzed or artificial saliva [34]. The acquired pellicle forms a protein‐rich diffusion barrier that slows acid penetration and modulates lesion progression [39]. Because salivary replenishment and pellicle reformation do not occur under continuous immersion, the erosive effects measured in this study likely represent an upper‐bound estimate. Future in situ and clinical studies incorporating salivary and pellicle dynamics are therefore needed to more realistically assess the erosive potential of RTDs [30].

Despite extensive evidence regarding acidic soft drinks, the erosive potential of RTD alcoholic beverages remains insufficiently characterized due to their diverse and evolving formulations. By evaluating multiple RTDs with distinct chemical profiles using complementary analytical techniques, this study provides new data to an underexplored research area and supports the development of future in situ and clinical investigations.

## 5. Conclusions

The RTD alcoholic beverages tested exhibited significant erosive potential, causing enamel softening, increased Sa, and measurable SL. Beverages with higher TA, stronger *β*, and organic acids such as citric and malic acid produced the greatest damage. Although in vitro conditions provide an upper‐bound estimate of erosion, the consistently large effect sizes indicate that beverage composition is the primary determinant of enamel degradation. Given the growing popularity of RTDs, greater awareness of their potential impact on dental health is warranted.

## Disclosure

All authors have read and agreed to the published version of the manuscript.

## Conflicts of Interest

The authors declare no conflicts of interest.

## Author Contributions

Conceptualization, methodology, validation, investigation, resources, data curation, visualization, supervision, and project administration: **Marcel Alves Avelino de Paiva**, **Paulo Vitor de Souza Silva**, **Anderson Gomes Forte**, **and Andressa Feitosa Bezerra de Oliveira**. Formal analysis: **Juliellen Luiz da Cunha**. Writing – original draft preparation, writing – review and editing: **Elizabeth Barreto Galvão de Sousa**. Final editing: **Marcel Alves Avelino de Paiva**, **Paulo Vitor de Souza Silva**, **Anderson Gomes Forte**, **Andressa Feitosa Bezerra de Oliveira**, **Juliellen Luiz da Cunha**, **and Elizabeth Barreto Galvão de Sousa**.

## Funding

This research received no external funding.

## Data Availability

The datasets used and/or analyzed during the current study are available from the corresponding author upon reasonable request.
